# Laparoscopic resection with organ preservation of recurrent well-differentiated retroperitoneal liposarcoma in long-term disease-free survival: a case report

**DOI:** 10.1093/jscr/rjaf572

**Published:** 2025-07-28

**Authors:** Seitaro Nishimura, Yohei Kurose, Rentaro Doi, Toru Noso, Naoko Miura, Wataru Ishikawa, Norihisa Takakura

**Affiliations:** Department of Surgery, Fukuyama City Hospital, 5-23-1 Zaocho, Fukuyama, Hiroshima 721-8511, Japan; Department of Surgery, Fukuyama City Hospital, 5-23-1 Zaocho, Fukuyama, Hiroshima 721-8511, Japan; Department of Surgery, Fukuyama City Hospital, 5-23-1 Zaocho, Fukuyama, Hiroshima 721-8511, Japan; Department of Surgery, Fukuyama City Hospital, 5-23-1 Zaocho, Fukuyama, Hiroshima 721-8511, Japan; Department of Surgery, Fukuyama City Hospital, 5-23-1 Zaocho, Fukuyama, Hiroshima 721-8511, Japan; Department of Surgery, Fukuyama City Hospital, 5-23-1 Zaocho, Fukuyama, Hiroshima 721-8511, Japan; Department of Surgery, Fukuyama City Hospital, 5-23-1 Zaocho, Fukuyama, Hiroshima 721-8511, Japan

**Keywords:** well-differentiated retroperitoneal liposarcoma, extended resection, tumor resection

## Abstract

Despite a favorable prognosis, well-differentiated retroperitoneal liposarcoma (WDLPS) recurs frequently. Although extended resection is advocated, its survival benefit is unclear and organ-preservation may be considered. A 46-year-old woman underwent hysterectomy, left salpingo-oophorectomy, and retroperitoneal tumor resection for a pelvic mass diagnosed as WDLPS. Three years later, a recurrence involving the rectum, pelvic sidewall, and vaginal stump occurred. Considering the well-differentiated appearance of the tumor on imaging, its unclear origin, potential multicentricity, lack of evidence supporting extended resection, and the patient’s desire to preserve the organs, we chose laparoscopic tumor resection with partial vaginectomy. We performed successful tumor removal with negative margins and transvaginal extraction using a specimen bag. Pathology confirmed recurrent WDLPS. The patient remained recurrence-free 6 years postoperatively. Laparoscopic organ-preserving surgery is a feasible minimally invasive option for treating recurrent WDLPS in appropriate patients. Individualized decision-making is essential because evidence for extended resection is lacking.

## Introduction

Retroperitoneal sarcomas account for 1%–2% of solid malignancies [1]. Retroperitoneal liposarcoma is the most common histological subtype, followed by leiomyosarcoma and solitary fibrous tumor [2]. Liposarcomas are classified into four histological subtypes: well-differentiated, dedifferentiated, myxoid, and pleomorphic, and well-differentiated is the most common. The 5-year survival rate of well-differentiated liposarcoma (WDLPS) is ~90%, but the local recurrence rate is 40%–60%, and a proportion of recurrences progress to dedifferentiated subtypes [3–5].

Even in recurrent cases, surgical resection is the first-line treatment if complete (R0) resection is deemed achievable [6]. Although extended resection including the adjacent organs may reduce the risk of local recurrence, high-level evidence is lacking. Surgical decisions must be tailored to tumor characteristics, extent of invasion, and the patient’s condition and preferences.

Here, we report a rare case of recurrent WDLPS that was successfully resected laparoscopically with preservation of the adjacent organs, resulting in 6 years of postoperative disease-free survival.

## Case presentation

**Figure 1:**
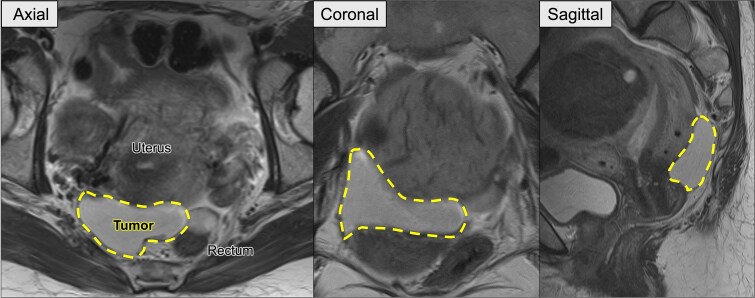
Preoperative pelvic MRI showing the recurrent retroperitoneal tumor (dashed line). Axial view: The tumor is located posterior to the uterus and anterior to the rectum. Coronal view: The tumor extends horizontally across the pelvic cavity. Sagittal view: The tumor is situated between the bladder and rectum, occupying the posterior pelvic space.

**Figure 2:**
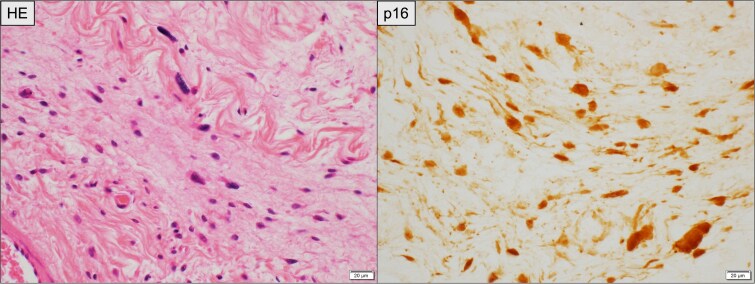
Histopathological findings of the primary retroperitoneal tumor. Left: Hematoxylin and eosin (HE) staining showing spindle-shaped atypical cells with abundant collagenous stroma suggestive of WDLPS. Right: Immunohistochemical staining for p16, demonstrating diffuse nuclear and cytoplasmic positivity in tumor cells. Scale bar = 20 μm.

**Figure 3:**
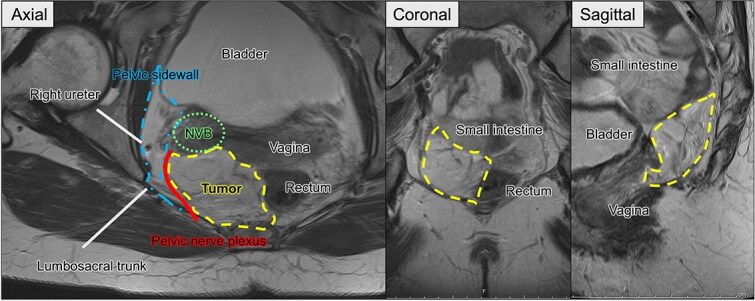
Preoperative pelvic MRI showing the extent and anatomical relationships of the recurrent tumor (dashed line). Axial view: The tumor is located adjacent to the rectum and vagina, abutting the pelvic sidewall (dash-dotted line), neurovascular bundle (NVB) (dotted line), pelvic nerve plexus (solid line), and right ureter. Coronal and sagittal views. The tumor is observed near the small intestine, rectum, bladder, and vaginal stump.

**Figure 4:**
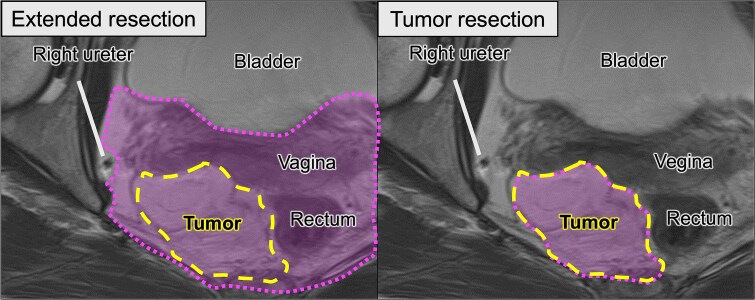
Comparison of surgical options based on preoperative MRI. Left (extended resection): The hypothetical resection area (dotted line) includes the tumor (dashed line), rectum, and vagina, corresponding to the multivisceral resection approach. Right (tumor resection): Planned organ-preserving resection where the tumor is delineated in yellow, and the surrounding organs are preserved.

The patient was a 46-year-old woman who previously underwent simple abdominal hysterectomy, left salpingo-oophorectomy, and resection of a retroperitoneal mass for uterine fibroids, left ovarian tumor, and pelvic mass. Postoperative pathological examination revealed WDLPS (Figs 1 and 2). She was followed as an outpatient; however, 3 years later, local recurrence was detected. The recurrent tumor was in contact with the rectum, small intestine, vaginal stump, pelvic sidewall, and fascia, from the anterior aspect of the sacrum to the levator ani (Fig. 3). Surgical options included extended resection, including adjacent organs, and tumor resection with organ preservation (Fig. 4). Resection with organ preservation was chosen for the following reasons:

1. The tumor origin was unclear. If it had arisen from the pelvic sidewall, R0 resection would have been difficult, even with an extended resection.
2. The possibility of a multicentric origin was considered. The risk of recurrence may have remained regardless of the extent of resection.
3. There was a lack of high-quality evidence. No prospective or large-scale studies have compared extended and conservative tumor resection.
4. Imaging suggested a well-differentiated subtype. Local control appeared feasible.
5. The patient strongly preferred organ preservation.

**Figure 5:**
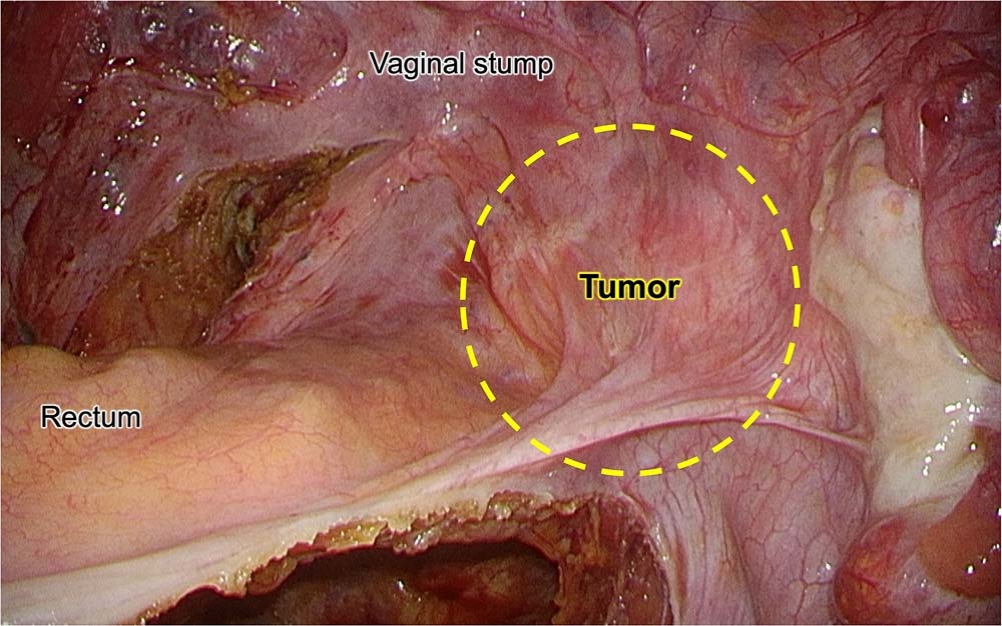
Intraoperative laparoscopic view showing the recurrent tumor (dashed line) located at the right lateral aspect of the rectum. The tumor is closely attached to the vaginal stump and surrounded by fibrous tissue, without a clear dissection plane.

Laparoscopy was selected given its advantage in accessing the deep pelvis. The tumor was adjacent to the right lateral wall of the rectum (Rb–Ra region) (Fig. 5). After mobilization of the sigmoid colon, rectal dissection was initiated from the dorsal and left sides. To avoid tumor exposure on the right, a lateral line was dissected along the medial aspect of the right internal iliac, allowing separation of the tumor while maintaining its attachment to the rectum. During the dissection, several structures, including the superior vesical artery, umbilical ligament, and pelvic nerve branches, were transected.

**Figure 6:**
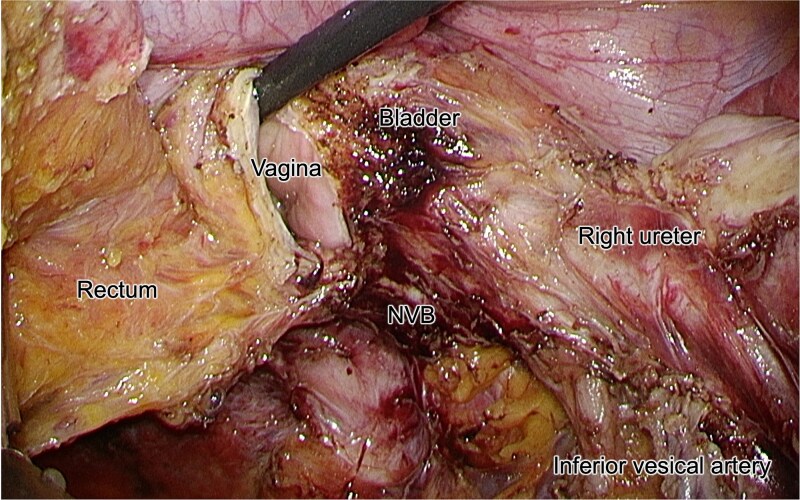
Intraoperative laparoscopic view following dissection of the tumor. The right ureter is isolated and preserved. The NVB, bladder, vagina, rectum, and inferior vesical artery are clearly identified.

No clear dissection plane was observed between the tumor and vaginal stump. Owing to the risk of ureteral injury, the right ureter was isolated using a vessel loop and was used as a landmark to determine the dissection line of the vaginal wall. Approximately 2 cm of the vaginal stump was resected along with the tumor, completing mobilization from the pelvic sidewall (Fig. 6). The tumor was separated from the rectum, with part of the mesorectum attached, and extracted transvaginally. The vaginal stump was closed using a continuous suture with 3–0 V-Loc™ (Medtronic, Minneapolis, MN, USA).

**Figure 7:**
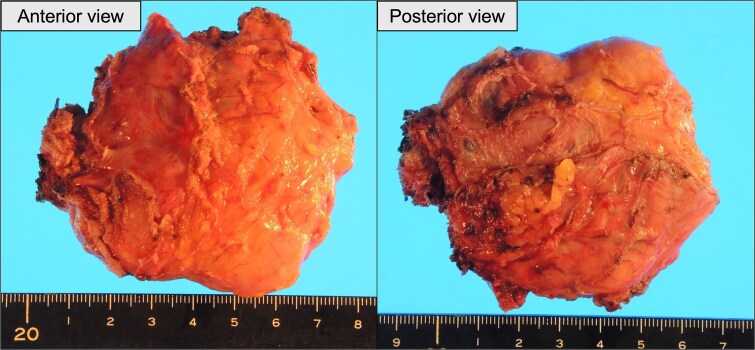
Gross appearance of the resected tumor. Left: Anterior surface. Right: Posterior surface.

**Figure 8:**
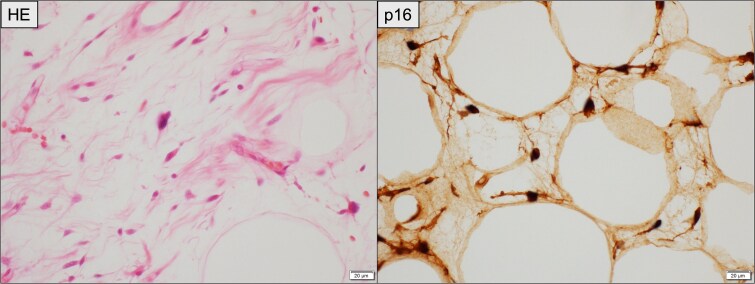
Histopathological findings of the resected recurrent tumor. Left (HE staining): Atypical spindle-shaped cells with hyperchromatic nuclei are observed within the fibrous stroma, consistent with WDLPS. Right (immunohistochemistry for p16): Tumor cells demonstrate diffuse nuclear and cytoplasmic positivity. Scale bar = 20 μm.

The procedure was described as a laparoscopic tumor resection with partial vaginectomy. The operative time was 5 h, 20 min, and the blood loss was 30 ml. The gross pathological (Fig. 7) and histopathological findings were consistent with those of recurrent WDLPS (Fig. 8).

The patient was discharged on postoperative Day 16. She has experienced lateral thigh hypoesthesia, neurogenic bladder, and constipation; however, she has remained recurrence-free for 6 years postoperatively.

## Discussion

This instance of recurrent WDLPS was successfully resected laparoscopically with organ preservation, resulting in a 6-year recurrence-free survival. Given the rate of local recurrence associated with WDLPS, this case offers valuable insights into organ-preservation and minimally invasive technique in selected patients.

Surgical resection is the first-line treatment for recurrent retroperitoneal liposarcomas when R0 resection is feasible [6]. However, the benefits of extended resection remain unclear. Compartmental resection has reduced the local recurrence rate from 48% to 28% [7], but the retrospective study had selection bias. Another group demonstrated that extended resection reduced recurrence, but did not improve overall survival (OS) [8]. A more recent analysis suggested that extended surgery did not prolong OS and might be detrimental to older patients [9].

Retroperitoneal liposarcomas may have a multicentric origin, and local recurrence may occur regardless of resection extent [10]. In this case, imaging suggested a well-differentiated tumor with relatively clear boundaries, and a conservative approach with minimal but sufficient margins was considered appropriate. The dissection line was defined laterally by the right iliac vessels and ureter, and medially by the mesorectum, allowing resection without tumor exposure. Extended resection would likely require rectal resection and stoma creation, which the patient wanted to avoid. Her preference for organ preservation played a substantial role in decision-making.

Laparoscopy was selected because of the deep pelvic tumor, and laparoscopic magnification offers superior visualization of critical pelvic structures. The ability to identify and preserve the internal iliac vessels, ureter, and part of the pelvic nerve plexus likely contributed to the avoidance of blood loss and intraoperative complications. Although reports on laparoscopic resection of liposarcoma are limited, Nomura *et al*. demonstrated the feasibility of laparoscopic resection of a 10 cm retroperitoneal liposarcoma [11].

Tumor extraction was performed transvaginally through the resected vaginal stump. This method reduced abdominal incision extension, postoperative pain, and risk of abdominal wall hernias [12]. After the planned partial vaginectomy, the specimen was safely retrieved through the opening using an extraction bag to minimize the risk of tumor spillage.

This case report has limited generalizability but underscores the need for further studies to determine optimal surgical strategies for recurrent WDLPS.

Our patient underwent successful laparoscopic resection of recurrent WDLPS with adjacent organ preservation and achieved long-term recurrence-free survival. In the absence of evidence favoring extended resection, individualized strategies based on patient preferences and intraoperative findings are essential.

## Data Availability

Data sharing is not applicable to this article, as no datasets were generated or analyzed in the current study.
